# The Rapid Development of Virtual Care Tools in Response to COVID-19: Case Studies in Three Australian Health Services

**DOI:** 10.2196/32619

**Published:** 2022-04-06

**Authors:** Kathleen Gray, Wendy Chapman, Urooj R Khan, Ann Borda, Marc Budge, Martin Dutch, Graeme K Hart, Cecily Gilbert, Tafheem Ahmad Wani

**Affiliations:** 1 Centre for Digital Transformation of Health University of Melbourne Melbourne Australia; 2 Bendigo Health Bendigo Australia; 3 Melbourne Health Melbourne Australia; 4 Austin Health Heidelberg Australia

**Keywords:** COVID-19, health system innovation, rapid development and deployment methods, remote patient monitoring, software development

## Abstract

**Background:**

News of the impact of COVID-19 around the world delivered a brief opportunity for Australian health services to plan new ways of delivering care to large numbers of people while maintaining staff safety through greater physical separation. The rapid pivot to telemedicine and virtual care provided immediate and longer term benefits; however, such rapid-cycle development also created risks.

**Objective:**

The aim of this study was to understand the sociotechnical aspects of the rapid-cycle development of seven different COVID-19 virtual care tools, and to identify enablers, barriers, and risks at three health services in Victoria, Australia.

**Methods:**

A qualitative, embedded, multiple case study design was adopted. Researchers from three health services collaborated with university researchers who were independent from those health services to gather and analyze structured interview data from key people involved in either clinical or technical aspects of designing and deploying seven different virtual care tools.

**Results:**

The overall objectives of each health service reflected the international requirements for managing large numbers of patients safely but remotely and for protecting staff. However, the governance, digital maturity, and specific use cases at each institution shaped the methodology and specific outcomes required. Dependence on key individuals and their domain knowledge within an existing governance framework generally enabled rapid deployment, but sometimes posed barriers. Existing relationships with technical service developers enabled strong solutions, which in some cases were highly scalable. Conventional project methodologies such as steering committees, scope, budget control, tight functional specification, consumer engagement and codesign, universal accessibility, and postimplementation evaluation were ignored almost universally in this environment.

**Conclusions:**

These three health services took a variety of approaches to the rapid-cycle development of virtual care tools to meet their urgent needs for triaging and remote monitoring during the first year of the COVID-19 pandemic. Their experiences provided insights into many social and technical barriers and enablers to the development of virtual care tools. If these are addressed proactively, they will improve clinical governance and technical management of future virtual care. Some changes can be made within individual health services, while others entail health system policy reforms. Enhancing the environment for virtual care tool design and implementation now will yield returns not only during future health emergencies but also in many more routine care settings.

## Introduction

The COVID-19 pandemic overwhelmed health resources in many countries. To handle unprecedented health risks and patient loads, health systems rapidly changed their models of care. In a matter of weeks, acute care health services around the world developed web and mobile applications to triage confirmed or likely COVID-19–infected patients (including their own health workers) to the most appropriate location, including outpatient testing clinics, hospital admission, in-home quarantine, or a dedicated isolation facility. Digital tools also enabled the hospitals to maintain clinical oversight of each remote patient through daily or more frequent contact (voice, text message, email, or other online service) and through the use of patient self-assessment tools to monitor symptoms (eg, temperature, heart rate, oxygen saturation, and respiratory rate). The term “virtual care tools”—including remote monitoring and tools (eg, [1])—describes this range of digital health interventions that track, monitor, assess, and manage decisions about care by health care workers and their patients when they are not colocated [2].

Health services had to make major decisions rapidly about their priorities for innovating with this range of virtual care tools. Some aimed at initial screening or triage of the general public or hospital staff, such as self-screening questionnaires (eg, [3-8]) and even chatbots [5]. Some aimed at follow-up of COVID-19–positive low-risk patients discharged from hospital, such as by providing them with pulse oximeters, thermometers, or similar digital devices; these innovations are described in an increasing number of articles (eg, [3,9-19]). Many virtual care solutions included web platforms with functionality for patients to report daily physiological data and symptoms of concern; if these data fell outside predetermined levels, suggesting deterioration, an automated message alerted the clinical team (eg, [10,11,15]). Other so-called “virtual ward” initiatives (reviewed by [18]) used technologies ranging from telephone calls to patient monitoring apps to capture the details of symptoms and personal data.

Some virtual care initiatives leveraged technologies already implemented in a health service, such as using or adapting existing electronic medical record (EMR) functionality to develop integrated tools for screening, triage, generating treatment order sets, remote monitoring of patients at home, and using health data effectively (eg, [5,6,10,12,14]). The implication is that patients were already enrolled in a health service EMR or could be enrolled readily in the EMR as necessary. Similarly, existing telehealth and video health applications were the foundation of some COVID-19 screening initiatives [20]. Other virtual care initiatives required novel technical solutions. Apps were developed for self-triage [4], for symptom grading [9], for reporting daily signs or to initiate a teleconsultation if a patient were concerned [11,14,17], or for use within the hospital emergency department [8]. Patient registries were established to identify, track, and monitor at-risk patients [12,20]. Purpose-built dashboards and analytics tools were used in some sites [6,9,10,20,21]. Survey software was also used for screening questionnaires [22] and for symptom tracking [17]. Continuous virtual monitoring infrastructure was adapted to monitor COVID-19 inpatients in negative pressure rooms and emergency department screening tents [20].

Design, development, and deployment of virtual care tools, which otherwise might take years to progress through research trial phases and clinical approval of software as a medical device (eg, [23]), were fast-tracked or short-circuited by the health care crisis. Few health services had time and even fewer had digital health expertise to attend to standards of technical development [24] or standards of clinical evidence [25]. Reviews published in late 2020 [18,26,27] summarized studies of the rapid deployment of digital technologies to cope with the COVID-19 pandemic in the early months of 2020, largely in the United States and Europe, most with fewer than 1000 participants. A case example in the University of Washington health care system [21] illustrates clinical informatics experts’ involvement in supporting the clinical management of COVID-19 patients, and highlights the rapid governance and change control mechanisms that enabled this rapid shift. Commenting on the difficulty of data exchange within and between countries, O’Reilly-Shah and colleagues [28] proposed rapid development and implementation of data standards to overcome fundamental barriers to a data-driven response to the risks posed by pandemics. Likewise, Lenert and McSwain [29] argued for wider informatics innovations to enable electronic health record data from health systems to flow into collaborative public health data repositories.

Many sociotechnical aspects of developing and implementing virtual care tools in these circumstances—decisions about hardware and software, clinical content, human computer interaction, people, workflow and communication, policies and procedures, laws and regulations, and system-level monitoring [30]—have not yet been described in detail. Therefore, in this study, we examined, from a sociotechnical perspective, rapid virtual care tool innovations in three health services, and consolidated the lessons they learned in the first year. In particular, we addressed the following two questions: (1) which virtual care approaches were chosen and how were they designed? (ie, hardware and software, clinical content, human computer interaction, clinical workflow and communication), and (2) how was innovation influenced by factors internal and external to the organization? (ie, policies and procedures, laws and regulations).

The pandemic affected Victoria, Australia, from the time it was first declared by the World Health Organization (WHO) in early 2020. Health services began preparation for the transition to virtual care when the virus’ impact on China, Europe, and the United States became evident through clinical communications, social media, and traditional media. Victoria’s COVID-19 case numbers fluctuated over the course of the pandemic and regional variation in numbers due to intermittent lockdowns was also apparent. This variation had minimal impact on the need to prepare proactively for worst-case scenarios, even though the utilization of the applications did vary greatly based on actual regional case numbers and infection waves.

The aim of this study was to provide insight into organizational readiness for future virtual models of care or service delivery through close examination of the evolution of virtual care tools during COVID-19 at three health services in Victoria, Australia. Understanding barriers and enablers that can be addressed in policy can inform the streamlining of current practices, and can ensure that digital health responses to future health crises and future routine care are managed and governed optimally.

## Methods

### Setting

This research was performed under the auspices of the Centre for Digital Transformation of Health at the University of Melbourne to address the need to facilitate cross-institutional learning during the pandemic [31]. The study took place at three health services in the State of Victoria, Australia. Austin Health (site A) and Melbourne Health (site C) are major metropolitan hospitals providing acute care and community services, and Bendigo Health (site B) is a major rural health alliance with 17 partner services.

### Ethics Approval

The Royal Melbourne Hospital (part of site C) is the designated state-wide provider for quarantinable diseases. Melbourne Health Human Research Ethics Committee approved this study (HREC/67522/MH-2020; October 16, 2020); this ethics approval was recognized by the other organizations involved, and governance authorization was obtained for each participating site.

### Design

This research used a qualitative, embedded, multiple case study design whose value has been demonstrated in previous health services research (eg, [32-34]). Researchers from the three health services collaborated with university researchers who were independent from those health services to initiate the project (project-managed by researcher URK), and gather and analyze data from people who had been involved in either clinical or technical aspects of designing and deploying the virtual care tools that were the focus of study: Austin Health community self-assessment platform [35], Austin Health COVID-19 symptom monitoring system, Austin Health COVID-19 symptoms management tool, Bendigo Health teamplay myCare Companion “Pandemic” [36,37], Royal Melbourne Hospital Home Monitoring [22,38], Royal Melbourne Hospital Screening Clinic tool, and Royal Melbourne Hospital COVIDCare. Table 1 describes the virtual care tools’ functions and features.

**Table 1:** Virtual care tools’ functions and features.

Health service	Tool type	Short description	Hardware and software requirements	Location of potential or current patient
A	Screening app (members of the public)	App that issues the patient or visitor with a QR code after they identify themselves and respond to COVID screening questions	Internal: internet connectivity, accessed through smartphones or web portal	In hospital
A	Screening app (staff)	Web page form in which staff identify themselves and their workplace and answer COVID screening questions	Internal: internet connectivity, accessed through smartphones or web portal	In hospital
A	Home monitoring	Patient routing self-assessment and triage app	Internal: Microsoft Azure cloud data storage; external: audio recording of breathlessness, pulse oximeters. Internet connectivity: smartphone	Out of hospital
B	Home monitoring (digital)	Patient routing self-assessment and triage	Internal: Amazon Web Services using Australian cloud data storage; external: pulse oximeters, digital thermometers. Bluetooth capability and internet connectivity on smartphone or tablet computer	Out of hospital
B	Home monitoring (with manual option)	Patient monitoring at home using manual and electronic Excel forms for self-assessment	Internal: Amazon Web Services using Australian cloud data storage; Microsoft Excel; external: internet connectivity. Computer or tablet (to read and input into Excel) or print capability to manually enter form	In hospital and out of hospital
C	Screening app (members of the public)	App that issues the patient or visitor with a QR code after they identify themselves and respond to COVID screening questions	Internal: Local data servers; external: internet connectivity. Smartphone with basic functionality: web browser–enabled and/or text message receiving–enabled	In hospital and out of hospital
C	Home monitoring	Patient monitoring at home for self-assessment	Internal: Local data servers; external: pulse oximeters, tablet computers with internet connectivity, or smartphone with internet connectivity/text message receiving–enabled	Out of hospital

### Interview Protocol

A semistructured interview protocol was developed based on the WHO digital health monitoring and evaluation guidelines phases 1 and 2 (prototyping and piloting), which focus on feasibility, usability, and efficacy [39] (see Multimedia Appendix 1). The questions sought individuals’ descriptions and observations of the process of developing and deploying the virtual care tools at each health service, including what clinical and technical features were prioritized and how the tools functioned in early stages of actual use.

Three site-based researchers who had lead roles in tool development at their site (GH, MD, MB) were interviewed themselves, and also nominated additional people to be invited for an interview by a university researcher (TW). Subjects for the interview were identified on the basis of their key roles in information technology (IT) and informatics or their clinical pivot to virtual care in the case of emergency medicine, respiratory medicine, or infectious diseases staff members. Participation was voluntary; all who were nominated agreed to participate given the strategic nature of the work (N=13, Table 2). In health services that developed more than one tool, interviews with staff who had been involved in development of each tool were conducted separately and data about each tool were collected separately. The interviews were conducted between December 2020 and March 2021 via video conference and were audio-recorded. They occurred after the tools had been deployed and significant numbers of cases had been processed. The interviewees therefore spoke with that experience in mind.

**Table 2:** Interview participants

Health service	Position
A	Clinical lead for virtual care tools/infectious diseases physician
A	Contracted external programmer
A	Director of information technology services
A	Chief Medical Information Officer (site-based PI^a^ in this study)
B	Director of Nursing
B	Executive Director of Information Services
B	Nurse Unit Manager (Admissions)
B	Registered Nurse (Psychiatry/Admissions)
B	Clinical lead, Integrated Care Services (site-based PI in this study)
C	Emergency physician/clinical lead (site-based PI in this study)
C	Assistant Manager, Nursing (Emergency)
C	Contracted external programmer
C	Emergency research director/senior physician

^a^PI: principal investigator.

### Analysis

Analysis of the interviews followed the 7-step process of the framework method, which provided a structured and systematic approach to analyze data, while also providing the necessary rigor required in qualitative research [40]. First, the interviews were transcribed with the aid of an online voice-to-text transcription service, and interviewees reviewed the transcripts for accuracy. Second, the interviewing researcher (TW) worked with two experienced qualitative researchers from the university (AB, CG); the three familiarized themselves with the transcripts, and TW annotated them with contextual notes that he had made during interviewing. Subsequently, the three researchers began the process of coding, independently analyzing and coding the first three interview transcripts using NVIVO software. They used WHO digital health monitoring and evaluation guidelines to characterize the tools deductively; they also performed inductive coding to characterize the interviewees’ comments thematically and to ensure that no themes were missed. The inductive approach used the open coding method, followed by constant comparison to refine the themes. The three researchers met regularly to discuss the codes, which underwent several iterations until they reached agreement on a working analytical framework. This framework was then used by AB and CG to code the rest of the transcripts. They aggregated comments at the level of each health service to reduce individual participants’ public identifiability. Thereafter, they sought corrections and clarifications on matters of fact from the principal researcher at each health service. All codes with relevant illustrative quotes were exported to a spreadsheet in the form of a framework matrix to summarize the data. The commissioning and coordinating university researchers (WC, KG) then applied Sittig and Singh’s [30] sociotechnical lens to review and organize the data into themes and subthemes for reporting. The three site-based researchers (GH, MB, MD) worked iteratively with the university researchers to reach consensus among all authors on the interpretation of the data and the implications for the Discussion section of the paper.

## Results

### Which Virtual Care Approaches Were Chosen and How Were They Designed?

Three priority aims for developing virtual care tools were described: reducing exposure (of staff and patients noninfected with COVID-19) by reducing admissions (including emergency department attendances), efficiency, and patient experience. See Textbox 1 for representative quotes for each aim.

The content of virtual care tools built by all three health services incorporated state government triage and management criteria [41]. Department changes and updates to their official case definitions were progressively incorporated into the screening and triage tools of the respective health services. Tools included risk stratification questionnaires relating to the general wellness of the patient (“How do you feel?”) and screening information related to travel and general symptoms (eg, headache, fever, diarrhea, cough, sore throat, and shortness of breath). Specific physiological aspects of heart rate, blood pressure, oxygen, and temperature were included in home monitoring for self-assessment, but patient data were collected in more than one way, using both digital and manual means and depending on the home context.

**Representative quotes reflecting the three priority aims for developing virtual care tools.**

- **Reducing exposure by reducing admissions**

“What we needed to do in the first instance was to make our staff as safe as possible to reduce the potential spread of the virus…The design [was] around two things: keeping patients away from the hospital - because we didn’t want to be overwhelmed, and we needed to keep the staff safe so that we had a workforce that was able to do the work... [also] Being able to give people up-to-date information and to have a screening tool, that could triage patients, to stop them from coming to the hospital, to go to centers that were closer to their home. So that we could just bring the sicker ones and the ones that needed our care.” [site A]

“The context was that we were asked to start a virtual home team which was looking after COVID-positive patients who were in the community...The key users that we targeted were COVID-positive patients who didn’t require hospitalization. The whole idea of this home monitoring is to prevent frequent presentations. That precludes the need for a lot of people to actually come into hospital.” [site B]

- **Efficiency**

“There's been a huge efficiency bonus—we could never have triaged that number of people, if they’d all physically come up and lined up in the car park, it would have been impossible. And the amount of data that’s been assessed would also have required hundreds, tons and tons of staff if we’d had to do it on fax or paper or any other technology. So it’s been very efficient for the hospital because the number of staff that have had to be deployed actually to managing this triage and a follow-up process has remained, I think, at three or four which we could never, ever have possibly done if it had been a more manual process.” [site A]

“Our senior emergency clinicians stepped back, asking, ‘What's going to happen when we get a planeload of potentially exposed travelers from overseas who are going to land in Melbourne and arrive at the emergency department all at once for screening? They’re probably all well. They’re all potentially infectious. How are we going to manage this?’ It became obvious to us that we would need to do something different fairly quickly.” [site C]

- **Patient experience**

“Patients are able to isolate within their own home yet feel like somebody’s watching them and monitoring them. There is a physical benefit because they can be in an immediate intervention if something is noticed to be wrong.” [site B]

“We want people to arrive in hospital just at the point in their illness when we can make a difference, and to leave when we can no longer make a difference.” [site C]

Virtual care tools’ functions are described in Table 1. All three health services provided home monitoring kits with digital devices such as pulse oximeters and thermometers and recording devices for breathlessness. Patients were provided with options for reporting their data; for example, they could manually record their oxygen levels measured using pulse oximeter devices and send the data via text message, or they could report to the triage team by phone call; tablets for connecting to web-based virtual care tools were provided to patients who had the capacity to use them. A variety of patient interfaces were described:

…can be configured to send the text to a carer, not just the patient. We do have knowledge of whether it’s a carer-based or a patient-based response.

site A

…if someone had difficulty utilizing the system, then we’d find someone else to add the numbers into the system, or they would phone us with the numbers and we’d enter their data on the system.

site B

If you're coming to a screening clinic…you could have six QR codes, with six languages and if you speak Italian, you just click the Italian one and the form will be sent to you in Italian and the information will go back in whatever format we want it to go to…in the screening clinic [we] have a couple of tablets or iPads, that can be handed out to people while they’re in the queue, to fill in the information

site C

Data management solutions varied from sophisticated cloud applications to Excel spreadsheets. Each health service developed local clinical workflows for reporting data, and in most cases for triaging respondents on clinical needs. Extraction of reportable information to the State Department of Health was enabled but had variable manual dependencies. No common clinical terminology (eg, SNOMED CT [Systematized Nomenclature of Medicine-Clinical Terms]) was adopted; however, Department of Health requirements mandated reasonably consistent word forms. How these were represented in underlying data structures was not consistent across sites. Data integration and systems interoperability were considered but were also foregone for the sake of expediency.

Generally speaking [the solutions] are quite separate and sole-purpose. Where relevant, data has been shared and there’s interoperability. Integrates with the contact tracing solution so we can monitor potentially exposed or at-risk staff for symptom. No integration with the [state government] contact tracing system.

site A

We know that integration is a key thing for us from a user point of view.

site B

It doesn't integrate directly into the EMR.

site C

Data privacy and security measures were implemented through local processes at each site. A participant from health service A noted:

Health is a very legislated and regulated environment. There were standard operating procedures under which this was all done…For example, we didn’t collect date of birth, just the age; not full addresses, just postcodes. So these principles were built into the apps

…a role-based approach to ensure that only certain people are allowed to see certain data; encrypted link sent to the patient or their carer

Platform choice was directed by ascertaining that data were stored in Australia on secure facilities, governed and secured by their own staff. Tools provided on browser interfaces relied on secure socket layer encryption. Health service C noted “Hopefully the robustness of the REDCap [Research Electronic Data Capture] platform is providing a layer of security.” Strategies such as segmentation of data sets were used to keep screening records for health services staff separate from reports for patients and visitors at the sites; health service B commented on the potential for staff to become patients themselves: “Because of staff privacy issues and confidentiality, [information] was segmented.” All used two- or three-factor authentication steps for patients to access portals or for staff accessing dashboards from their own devices. IT staff also reported using penetration tests and running vulnerability assessments on their systems.

### How Was Innovation Influenced by Internal and External Factors?

All three health services had to create new project teams and use a rapid cycle design (“agile”) philosophy [42], necessitated by the clinical requirements and human resource management prescribed by public health officials and local infection control experts. Projects were built on existing staff capability, assisted by contract programmers or developers, led by senior IT and clinical staff who were familiar with privacy and security requirements and who had the authority to proceed. The agile project team had responsibility for each of the rapid-cycle applications. This was a shared experience at each site. There were daily design, build, test, and adapt meetings to ensure the fastest possible deployment. Once a stable base system was established, additional functionality was added in a similar rapid-cycle format. Traditional considerations such as final functionality specification, design, tender, contract, and project governance largely were suspended. In applications where internal clinical staff were the end users, there was significant informal engagement in the design and build phases, including testing functionality and wording. This was not the case where the end users were consumers. User acceptance testing was incorporated into the rapid-cycle development framework. Postimplementation evaluation was mainly informal and based on functional user acceptance and utilization numbers. In addition, some of the virtual tools were adopted by other Victorian health services for use in the pandemic or were modified for use in other clinical settings.

The solutions deployed at each site were dependent, in part, on existing infrastructure, agile teams, applications, and relationships. Victoria differs from other states in Australia in having a decentralized system where each health service operates under its own board, following objectives and outcomes set by the State Department of Health. Each health service in this study uses a different EMR (site A, Cerner Millennium; site B, Intersystems; site C, Epic). These are at various stages of implementation, but fall within intermediate to high bands of digital health maturity, that is, levels 3-6 of the Healthcare Information and Management Systems Society’s Electronic Medical Record Adoption Model. At each site, access to informatics and IT staff to rapidly build and deploy COVID-19 applications was not straightforward; they were generally fully engaged in other projects or intensive adaptations to prepare for COVID-19 in business-as-usual operations. However, internal development was essential due to the short timeframe for decision-making, without specific direction from state health authorities or market-ready software. Where local staff could not be redeployed, additional contract staff with relevant computing expertise were engaged through existing relationships with external consulting companies for this purpose, and all external service providers contributed substantial goodwill to these projects.

Site A had standing multiyear master services agreements in place that enabled application development within enterprise software solutions. Health service A is also moving toward procurement panels similar to larger-scale state procurement panels to reduce the effort and time to project commencements. In this case, there was a continuity of mutual understanding and trust that the intent of organizational policies and procedures would be observed during the rapid-cycle deployment. Health service A had been building knowledge and capability of artificial intelligence, machine learning, and natural language processing in their Microsoft Azure tenant together with external developers familiar with that platform. This platform allows flexibility in application selection and is highly scalable. As this relationship already existed, it was used to rapidly build and scale the applications. Together with the product developers, they agreed on a base application and rapidly designed electronic forms for a symptom reporting web-based application hosted on the health service’s Microsoft Azure cloud.

Similarly, site B leveraged an existing relationship with the commercial product developers of their patient monitoring platform. Prior to the pandemic, health service B was in discussion with Siemens about a chronic disease management home monitoring tool and app. Senior management approval was granted to proceed to pilot on this basis. The clinical team responsible for chronic disease home monitoring became the group that took on the monitoring of COVID-19 patients.

Site C used an existing online research data system (REDCap) to rapidly build their capability. The clinical requirement at health service C was determined by local clinicians to manage expected high numbers of attendances at the emergency department. Development was driven by emergency clinicians and the tools were programmed in the service’s established REDCap electronic data capture system [43]. REDCap was already approved for clinical research use; its three virtual care tools (patient screening, staff screening, and home monitoring) were granted a waiver of ethical clearance, as a quality improvement program. This system was highly dependent on two clinicians not normally involved with IT or EMR development to maintain service and alter software in a rapidly evolving data content environment.

Three enabling factors were noted to accelerate development of the tools in all three health services: having access to external IT developer expertise, close collaboration between internal clinical and technical staff and external developers, and strong support from senior management. Comments from site C summed up the organizational obstacles to be overcome, “the hospital bureaucracy committees, red tape permission, all of that,” and the enabling characteristics, “it helps to have someone in the organization with the ability to question what is the outcome that’s needed, and you need a small group able to work intensively on a solution that matches this.”

Being enabled by working within an integrated health information environment was also expressed, but as an unmet need:

Victoria needs […] to have a single EPR [electronic patient record] or EMR so that these things would all link uniformly into the same platform…

You need adequate architecture linked to systems and the workforce.

Companies want to deal with you […] and they don't have a vision about an integrated, a fully integrated connected care model.

site B

Enabling funding was deemed secondary to rapid and effective deployment at the metropolitan health services, as those sites faced substantially increasing COVID-19 case numbers. The regional and rural health service (site B), with a lighter case load, voiced a greater sense of the financial resource limitations:

You can have whatever you want, provided you’re prepared to pay for it.

A major intervention like this requires a considerable amount of development. If you’re buying something off the rack, so to speak, to tailor it to the needs of your particular area takes time and effort and work, which again, equates to money at some point, but it's resourcing and availability of resourcing.

Social barriers to design and implementation of the virtual care tools emerged at all three sites. The prime concern was the digital divide affecting some groups of health service users:

There were concerns about accessibility for the non-English language speakers and the elderly, or the “digitally challenged,” if you like.

site A

We initially looked at connected oximeters and trying to capture that data automatically. What we found was that that seemed to be a barrier of entry because the patient would then have to figure out how to connect at home and go through that process.

site A

We’re contacting people and finding out they didn’t have computers or didn’t understand computers.

site B

The cohort that we had with these chronic complex issues were elderly patients. So there were very few patients who were computer literate and very few patients who even had phones and computers.

site B

You’ve got to look at the user interface to see whether it’s practical and whether it provides a product that they can utilize fairly intuitively.

site B

A little bit more challenging for the uptake by the consumers because 12 months ago, a lot of us didn’t know what a QR code was. But we built in some education around that…installed some big posters on the wall on how to access a QR code.

site C

To sum up, key results reported here can be mapped against the eight dimensions of a sociotechnical framework summarized in Textbox 2. Multimedia Appendix 2 presents additional interview quotes that illustrate these sociotechnical themes.

**Dimensions of the sociotechnological framework.**

- **Hardware and software**

The sites worked around rather than through their electronic medical record systems; their solutions to store and process patient data varied from local servers to cloud services. Solutions assumed that the majority of people had ready access to internet-connected phones or tablets.

- **Clinical content**

Clinical content was structured in the most expedient way and prioritized to the essential elements for two decision support scenarios: triaging emergency attendance at a hospital and monitoring home-based care remotely.

- **Human-computer interaction**

An array of novel reporting and analysis interfaces was assembled for staff, with little concern for their overall user experience under the circumstances. Unfamiliar information interfaces such as QR codes, oximeters, and teleconsultations became normalized for health service consumers.

- **People**

Small teams of digital health champions undertook extraordinary development efforts. Given infection rates, both health service consumers and health care workers were likely to be patient “end users” of the solutions.

- **Clinical workflow and communication**

Initiatives at these three sites went beyond the scope of routine care and practice. They contributed efficiently and effectively to preserving health workforce capacity and preventing health service overload.

- **Internal policies and procedures**

Routine business processes for planning and implementing digital innovations did not run at the speed required by the health crisis. Innovation arose in organizational cultures where there was a foundation of trust in key staff and industry partners.

- **External policies and procedures**

In the absence of shared, centralized management of hospital information systems, the sites in this study mobilized informal communities of practice. This approach to sustain and extend the use of virtual care solutions beyond the health crisis will depend on shifts in digital health regulation and resourcing.

- **System monitoring**

The sites in this study have committed to formative evaluation of the development and piloting stages of their virtual care solutions in the form of research reported in the present paper. The World Health Organization staged evaluation model referenced here carries the expectation of further monitoring of solution performance and outcomes over a longer term of implementation.

## Discussion

In summary, all three cases showed priorities, issues, and outcomes similar to those in the COVID-19 virtual care literature around the world: rapid development and iteration, staff and community safety through distancing and virtual care, scalability and efficiency, uncertainty, and constraints of business-as-usual models. The ability to rapidly leverage existing infrastructure and relationships proved critical in each case, even though the technical responses varied considerably. None of the sites used their EMRs as the primary digital tool, largely due to the administrative difficulty of enrolling so many new patients formally into the EMR. The tools that were developed enabled consumers to self-register and hence reduced the administrative burden, which is known to be significant in other jurisdictions where an EMR was used (A Ritchie, Chief Medical Information Officer, Sydney Local Health District, Royal Prince Alfred Virtual, personal communication, May 13, 2021).

Governance maturity was just as important as technical maturity; organizational dependence on trusted employees to drive these projects in a responsible and defensible manner was critical to timely, successful application deployment. Maturity implies that the organizations had developed sufficient internal processes and policies, so that responsible staff could rapidly deploy within those principles without necessarily following the operational and committee procedures, which would have taken too long. Informal sign-offs were more often the case, even though one health service found that there was a tension between formal and informal processes. Projects at all three sites had a high level of risk because of their critical dependencies on a few key actors. These were pressing factors in the pandemic setting, but these might not be as relevant in postcrisis or in less threatening situations.

Difficult choices needed to be made regarding time to deployment versus equity of access. All respondents noted that there were certain consumer groups who could not access these applications due to limitations in language, technical access, or skills. These issues were identified internationally, such as by Houlding et al [1], where barriers to the use of remote monitoring technologies included equity-related barriers (such as affordability of technology for users, poor internet connectivity, poor health and digital literacy), the need for good practice guidelines for use in clinical care, and the need for additional resources to develop and support new technologies. In our study, some access issues were mitigated by use of patient proxies and one health service enabled some multilingual content. Consumer consultation and codesign were noticeably absent as were formal evaluation and benefits realization. To overcome these issues systematically as virtual care tools become part of business as usual, health services will need to establish consumer engagement structures proactively and embed them in usual practice so that these ways of working with consumers are trusted and effective. Another victim of development and deployment speed was the failure to use recognized coding systems and terminologies in the apps design. There was congruence of clinical concepts, clinical triage criteria, and state government reporting requirements, which drove content in these apps; however, the underlying data structures are inconsistent and would require mapping to consolidate the data. This is another area where embedding capability into usual practice, specifically stronger informatics skills within health services’ IT teams, would provide benefits.

Health services have widely recognized the success of these rapid IT implementations and now have more confidence in enabling and sustaining virtual health care applications [3]. Although it is a limitation that consumer views have not yet been assessed in our study sites, we note that strong consumer satisfaction is reported in the literature (eg, [3,15]). Further developments would be streamlined by formalizing the enabling and success factors identified in this study and building in mitigations for the deficiencies (eg, consumer panels and multilanguage capabilities embedded in consumer-facing digital health applications). Formal partnerships and collaborations between health services and IT companies may offer a significant long-term benefit for in-house design and deployment and should have a legitimate place alongside the competitive project tendering mechanism. This is especially the case where enterprise platforms are used for related deployments.

The authors of this study brought our diverse experiences and perspectives as practitioners, technologists, administrators, and researchers to bear to reflect on the data that we highlighted in our Results section and on additional thematic data garnered in this study (as summarized in Multimedia Appendix 2). Our collective lessons learned about barriers, enablers, and suggestions for future work are captured in the recommendations we propose in Table 3 and Table 4.

**Table 3:** Recommendations for improvement in policy and practice, based on barriers experienced in rapid virtual care tools development (after Houlding et al [1]).

Barriers	Normal practice	Rapid cycle	Suggestions for future
Poor internet connectivity	Solution delivery platform designed for optimal functionality for the target population; multichannel delivery with “low tech” where possible	Solution delivery can take into consideration the availability, access, speed, and other requirements of applications depending on the project context. The latter can encompass pilot applications across any of 3G, 4G, 5G, and WiFi	Create a registry of available technology platforms for areas where optimal solutions are unavailable; these could be related to geography, topology, rurality, service outages, or cost of access
Low health literacy	Design with language optimized for target populations	Considerations often overlooked or unavailable in the project context due to emphasis on rapid prototyping without participation of a spectrum of service users	Create design templates for developers to utilize in specific low literacy populations
Low digital literacy	Design with “low tech” optimized for target populations	Considerations often overlooked or unavailable in the project context due to emphasis on rapid prototyping without participation of a spectrum of service users	Consider development of support models for users (eg, training, adoption support)
Need for quality, best-practice guidelines for use of remote monitoring technologies in clinical care	Project design and funding aimed to support optimal solutions	Utilize technology already available and approved; design interface around available devices	Development and maintenance of tool sets and guidelines for remote and home monitoring use
Lack of resources to develop and support new technologies	Project design and funding aimed to support optimal solutions, which may include new technologies where feasible	Rapid design of applications leveraging existing technologies, devices, and/or platforms. Innovation is often in the reuse of technologies to extend and/or enhance functionality	Structured simulation and validation frameworks for rapid-cycle development, testing, clinical trial, and deployment. Consider total cost of ownership (eg, unmeasured development costs, hosting costs). Manage human resources cost (eg, informal time of subject matter expert clinicians, technical developers’ time)
Equity-related unaffordability of technology for users	Scoping outcomes and target clientele; mitigations for identified consumers; alternate funding models or subsidies; design for least-cost technology; stratified interventions	Equity considerations frequently not addressed; 80/20 rules due to rapid prototyping process for majority service users; mitigations for descoped users may be considered in a subsequent evaluation phase	Identify and resolve long-standing equity and access issues so that standing solutions are available to be incorporated at short notice; build in multilanguage capability; develop policies and mitigations for equity access as part of business as usual; apps delivered if possible over multiple channels, including low-cost SMS and phone

**Table 4:** Recommendations for improvements in policy and practice, based on enablers experienced in rapid virtual care tools developments (after Houlding et al [1]).

Enablers	Normal practice	Rapid cycle	Suggestions for future
Governance: policies reflect required outcomes and describe allowable emergency and rapid-cycle processes and permissions framework	Review internal policies and ensure they reflect both business-as-usual and emergency situations to enable appropriate rapid responses	Existing policies describe acceptable process and outcomes in the absence of conformance with standing committee and approvals framework in defined circumstances	Identify “special needs and emergency” situations; review business-as-usual practice to reduce unnecessary delays
Master services contracts: reducing procurement delays with trusted providers	Individual projects defined, budgeted, and tendered; project management framework defined	Existing relationships leveraged to create short-term team with focused but flexible and evolving outcomes as external environment evolves	Establish panels of approved partners and consultants to enable rapid design and deployment, especially using existing enterprise solutions
Standing consumer working groups for rapid cycle codesign	Consumer groups engaged on project basis, often ad hoc	Consumer groups might be largely ignored in the rapid prototyping process and in participatory practices over the course of the rapid cycle	Establish panels of educated consumers who can contribute knowledgeably across all projects and be available at short notice; actively engage a spectrum of users and consumer organizations; cocreate a participation framework with a cross-section of consumers/service users throughout the life of the service, including options for training (eg, digital and health literacy)
Upskilling and enabling clinicians and subject matter experts to lead projects targeted at their specific issues (eg, predicting issues and rapid problem enunciation)	Clinician-led projects battle for priority and resourcing against “top-down” projects	Clinician-led and developed applications target local requirements using defined, secure, integrated platform applications; informal international clinical networks and peer review rapid publications flag concerns prior to official body pronouncements: lead the local curve	Training and enabling clinicians in supported platform applications (eg, Dynamics, Forms, REDCap^a^), reduces lead time and impact on core IT^b^/EMR^c^ applications teams. Clinical networks promote data conformance and spread of successful applications
Collaboration (clinical and technical) networks facilitate shared understanding and requirements for development, together with resource sharing	Organizational, cross-organizational, and professional and clinical networks advise on priority applications and consulted ad hoc regarding application selection and deployment issues	Existing networks should be convened as priority to coordinate and share resources to expedite planning development and implementation. Parochial variation should be reduced or eliminated	Convene, support, and sustain these networks as business as usual so they deliver benefits and are functional when required in emergency scenarios

^a^REDCap: Research Electronic Data Capture.

^b^IT: information technology.

^c^EMR: electronic medical record.

This study provides an in-depth qualitative assessment of the sociotechnical environment in three large Australian health services during the rapid deployment of staff- and consumer-facing COVID-19 applications. Its strength is in capturing the diversity of technology platforms and development models and sharing the reflections of key personnel across these three sites. Although the number of interviewees perhaps seems limited, in fact, all key decisions were made by those few people, in consultation with other key clinical groups. Where possible, clinical staff who both provided expert advice on form and content of the applications and who became key testers and users of the applications were interviewed. In terms of a full sociotechnical evaluation, the paper has limitations due to the focus on operational efficiency at a time-critical period in history. The availability of multiple cases located within the same state of Australia (Victoria) provided an opportunity to describe and analyze varied experiences of virtual care innovation within a national health care system.

During the initial period of the pandemic, state health policy makers were occupied in managing whole-of-system preparedness, state and national reporting systems and contact tracing systems, vaccination preparation, and quarantine. In Victoria’s decentralized public health system, each health service is responsible for implementing state policy in its own way, and this will be partly determined by prior decision making around EMR/paperless administration system selection, implementation stage, and other elements related to digital maturity. In a similar manner, hospital executives, who are often overwhelmed by preparations and hospital ward reconfigurations, staff protection and visitor policies, logistics, and supply chain challenges, provided support but not direction for those clinicians and EMR/IT experts charged with response and virtual care preparation. The state government provided a significant funding stream for COVID-19–related activities, including virtual care preparation. Expenditure allocation was authorized by department heads in consultation with finance department officials.

This context makes our results less comparable with other states in Australia where digital health strategy and implementation are more centralized. A limitation of our study is that we were constrained from including cases from additional major health services in Victoria, in part due to onerous, site-by-site research ethics and governance processes. Further work needs to be done on evaluation. In particular, we could not meaningfully interpret usage data to give a clear picture of immediate uptake of these applications, and we are not yet able to provide a full sociotechnical analysis of longer-term, scaled-up experiences or outcomes. Such evaluation research is the focus of another study.

Despite these factors, our findings are consistent with other reported projects of similar nature in the international literature. The presence of mature EMR and supporting IT infrastructure and governance models enables rapid development and deployment of digital health applications in support of new models of care. Preexisting contractor relationships at two study sites enhanced the capacity of those services to deploy rapidly. All of the relationships reported here underwent standard probity assessments when initially contracted (eg, fairness, avoidance of favoritism, hidden inducements); however, within these contracted relationships there was scope to modify and evolve service requirements within reason. The preexisting personal and technical engagement enabled the rapid-cycle design and development of the COVID-19 virtual care tools. Conventional governance models are essential for establishing normative behaviors, checks, and balances; however, emergency, rapid-cycle developments can be performed safely using the principles established by this governance without rigid adherence to committee structures and procedures. User acceptance of virtual digital applications described in the international literature is high, and the intent to support large cohorts of at-risk people and manage emergency service utilization has been demonstrated. Direct consumer involvement and codesign were not features of our study cohort, nor were these aspects comprehensively described in the literature about other COVID-19 applications. Applications supporting clinicians were more likely to have undergone some degree of codesign, with short-cycle deploy review and revision, simply because clinician users were in proximity to app developers.

In conclusion, we agree with Boyle and Henderson [44]: “As we move from a pandemic to an endemic state, delivery of care must also change to ensure this—and similar diseases—can be managed safely, alongside regular emergency care, within our departments and wider healthcare systems.” The findings of our study confirm that the pandemic has affected us in similar ways to the rest of the world and that our responses have been similar. Lessons learned elsewhere have relevance for Australia; it is clear that rapid deployment of triaging and remote monitoring technologies has occurred successfully in numerous countries and contexts. These findings support clinical recommendations, to governments and other funders, that structures and systems for developing virtual care tools should be strengthened in organizational and funding models. This will not only engender resilience in health emergencies but also has the potential to transform chronic disease management and routine clinical care to be more accessible and less costly and to reduce pressure on fixed health service infrastructure.

## Appendix

Multimedia Appendix 1 Indicative interview questions about virtual care tools.

Multimedia Appendix 2 Additional interview quotes illustrating sociotechnical themes.

## Abbreviations

EMR: electronic medical record
IT: information technology
REDCap: Research Electronic Data Capture.
SNOMED-CT: Systematized Nomenclature of Medicine-Clinical Terms
WHO: World Health Organization
