# Supplementary material for: The Rapid Development of Virtual Care Tools in Response to COVID-19: Case Studies in Three Australian Health Services
Source: JMIR Form Res. 2022 Apr 6;6(4):e32619. doi: 10.2196/32619 (PMC8993142; doi:10.2196/32619)
Supplement: Multimedia Appendix 1 [file formative_v6i4e32619_app1.docx]

*Multimedia Appendix 1***:** Indicative interview questions about virtual care tools

A. Context

What were the exact circumstances of developing the app?  For example, who/ when/ how  … came up with the idea? … contributed to the brief about what was required? … did the work of developing it? … reviewed progress on development? … approved the project?

Was there a full project brief or an agile approach?

What factors influenced the decision to partner / not in development, or to co-design?

Which health service roles / influences defined the service elements and data elements required for this app

What was the technical platform? E.G. EMR Vendor / Patient Portal / Web app / hosted internally / cloud tenant Web app Cloud tenant AI Redcap Other

What was the time from inception to go-live?

What stage of deployment is the app in now?  For example, is it at the stage of fully approved for clinical use, or a formal clinical trial, or a pragmatic trial, or a pilot study, or a working prototype?

Anything else you’d like to add about the background to development?

B. Users

Who are the intended users? For example, which patients? external patient enquiries / visitors to inpatients / COVID tested follow up patients / others

Which staff / staff groups monitor and respond to the information? – emergency, respiratory, infectious disease, etc. Anyone else?

In what digital format/s do users work with the intervention?  For example, a mobile app? / online survey? / kiosk? / dashboard?/ web portal? / other?  If it takes a different form for different kinds of users, please describe each. For example, patients use an app; clinicians use a dashboard.

Is there provision for language other than English, visual or other disability?

What software / platform / telecomms do users need, for the intervention to work?  For example: home computer running Windows 10; Android / iPhone ; 5G network; devices supported

Anything else you’d like to add about the intended users?

C. Functionality

What types of data are collected from a patient?   patient identifiers collected / assigned -

Patient Record Number - database assignment - random number - hashed ID - mobile phone number -other ( specify)

How are these data protected?  - Firewall -Encryption- Hashing- Not retained

What clinical data? Symptoms -Patient measured heart rate, respiratory rate, temperature

Device used to capture data? Provided by health service / other / patient wearable device / visual images captured from patient device / audio signals captured from patient device

D. Information flow

What types of clinical decisions does a staff member make?  Describe any algorithmic escalation or triage capability. Describe any AI / ML based analysis of individual patient data

What types of advice does a patient receive?  Clinical care advisory / proceed to this health service care location / proceed to other health service

What types of information about patients and clinical decisions are aggregated for review?  In-hospital / health department / other

Anything else you’d like to add about the functions and features of the virtual care tool?

E. Evaluation of development and deployment

Please provide details of the inputs that have gone toward developing and deploying it. For example: person-hours, contractor costs, communication costs, other.

What resource constraints or sources of resourcing especially affected the scope of work?

What would successful uptake look like?

Please provide details of uptake / usage. For example: actual users / levels of use compared to projected users, other aspects of usage

Please provide details of issues encountered during use. For example: number and types of issues observed / reported, other performance shortcomings

Please provide details of risk management. For example: extent of compliance with health data privacy and security requirements, other management / governance concerns for the health service

Has an app description (including nature of proposed use, data collected, proposed secondary data use, data protection and consent) been reviewed? By which organisational unit?

Is there a plan for a formal post-implementation review?
