# Supplementary material for: The Rapid Development of Virtual Care Tools in Response to COVID-19: Case Studies in Three Australian Health Services
Source: JMIR Form Res. 2022 Apr 6;6(4):e32619. doi: 10.2196/32619 (PMC8993142; doi:10.2196/32619)
Supplement: Multimedia Appendix 2 [file formative_v6i4e32619_app2.docx]

Multimedia Appendix 2: Additional interview quotes illustrating socio-technical themes

Theme 1: hardware and software:

- “*Keep it as technology-neutral as possible* [e.g.] *using web browser apps rather than iPhone or Android-specific apps reduced that development time and cost*.” *–* Site A, principal investigator.
- *“I think from an actual end user point of view, ease of access for devices and their ability to input information as required is something that we clearly want in the system. We want to make it as hands-off as possible, so that any device they're using is relatively automatic. Even if it means, for instance, that we've got purchased mobile phones that we're handing to people, so that they've just got it there…it's the link for us.”*  – Site B.
- *“We will use a better tool in the future … It'll be consistent across an application. And, we can control what data we collect, how we want to collect it, and maybe even do some smarts in the data collection in the background so it's prepared and ready - we don't have to worry about doing some unpacking at the other end.”* – Site B.

Theme 2: clinical content:

- *“Initially it was all about testing. How do we best streamline the process of testing people for COVID at a time where there was high community anxiety but also a relative lack of access to testing capabilities, not very many test kits, relatively slow turnaround on results? Trying to streamline that, so that the right people got access to testing, and in a timely fashion, without also then swamping our clinic with hundreds of people who didn't necessarily need a test. So that we could identify the individuals who were eligible, and facilitate them safely coming in. So, in conjunction with the IT developer, the first thing we did was develop that screening tool.”* – Site A.
- “*The person’s risk criteria were used in the* [visitor/outpatient/staff] *screening tool.* [In the home monitoring tool COVID-Care] *the patient’s comorbidities, previous medical history, symptoms and data from oximeter were used to prioritise follow-up care. It’s always up to the clinical team to make the final decision…we helped prioritise follow-up based on severity of the reported symptoms.” –* Site A.
- *“On 6 January 2020 we began electronic prompting by our triage nurses to screen if people presenting to our hospital had been in Wuhan recently…The first COVID case arrived in Australia around 17 January. On 27 January, when the first flights from Wuhan landed in Melbourne, the Victorian Department of Health and Human Services advised all passengers on those flights to be tested at our hospital emergency department.* *So we built a digital questionnaire… We had the ability to collect a whole lot of information about their symptoms and their exposures to work out whether they required swabbing or not… We adopted the [Victorian government’s] case definition of which symptoms and exposures to look for.” –* Site C.

Theme 3: human computer interaction:

- *“You've got to look at the user interface to see whether it's practical and whether it provides a product that they can utilize fairly intuitively. So, you've got to think about the elderly versus the young who are better on using their mobile phones, you've got to think about how comfortable they feel with even doing this telehealth, all the folks, even younger folk. Fortunately COVID has diminished that as a worry because people become very familiar with it and [are] so much more comfortable. And in fact, some of them even prefer to do it that way. So that's another layer. You need adequate architecture linked to systems and the workforce. The workforce: no one ever mentioned them in all this conversation. What's really behind all of this is digital health literacy and teaching people about digital health. Especially the technical issues, you know, what is an asynchronous telehealth consultation versus a synchronous one? What is machine learning even? What does it mean?* – Site B*.*
- *“The real innovation was, while [patients] were waiting to be seen, to get them to start filling out their own information. And so it was all done very quickly, [using] REDCap, which is the data collection tool. People could scan the QR code… back then, it was quite an innovative idea to have that [QR code] sitting in the ED, not only in English, but in six languages. People could scan it and then they would fill out their own information and their own details, be given some information, pushed to them, and then submit. And by the time they reach the clerk to be initially put in the system, a lot of that information was there and could actually be taken and pasted into the medical record. So it allowed a very rapid collection…” –* Site C.

Theme 4: people:

- *“The context was that we were asked to start a virtual home team looking after COVID-positive patients who were in the community…Not all of them needed home monitoring, but they were all monitored by us to a certain degree.* [That might be] *just a daily phone call or* [might have required] *a virtual monitoring situation.”* – Site B.
- *“Looking back…we were just trying to work it out as we went. So every time it expanded, changes were made, and it seemed much smoother. You can expand and get more out of what you're doing. But because we were doing it on the fly and sort of implementing it within that 24-hour period, that was challenging.”* – Site C.
- *“When I spoke to a few patients, what they were really happy about…is that someone was keeping an eye on them, because here are people that are COVID-positive, they're quite anxious, no one knows much about this virus, they're being sent home, because they're physically well, and yet, there was someone who was keeping an eye on them, monitoring them, even if it was mostly remotely and digital. And it just reassured them they were well. I guess the flip side of that is, we did have some people who got really sick, who started off okay and became unwell. And we were able to pick that up. We actually picked up a number of people who were probably in the early stages of deterioration, and bring them in.”* – Site C.

Theme 5: clinical workflow and communication:

- [For the remote monitoring tool] *“COVID-positive patients received one message each day, for 14 days.* [At first] *“People who weren’t positive were sent one message a day for 14 days, but then we reduced it to a message each second or third day for 14 days…*[Each message asked] *Tell us how you’re feeling? For operational use, staff could see the responses as they came in; for reporting purposes we had a daily extract.”* – Site A.
- *“…The [home monitoring] information was coming in every day; we had the information graph in front of us. The clinicians were looking for any significant changes that happened within the data. And having the physical data with the written observations, they're also then looking at what people felt because often what we would see is people would actually verbalize that they, for instance, were feeling unwell or that they didn't feel as good as they had yesterday before we saw a physical change...They would be looking at what the person was saying [and] the observations, to look at how that was comparing to what they had provided in previous days. And then obviously the physical vital signs as well.” –* Site B.
- *“Look, I think [remote monitoring] is sort of making people more self-manage because they are putting the information in, rather than us having to contact them regularly to see how they're going. With someone that's fairly stable, it would be good to just to have that; and have a quick look at what the vital signs are. And if they're doing okay [..] that stops you having to ring and make a 15-minute phone appointment or something. So it's probably a bit of a time-saver.”* – Site B.
- *“I think knowing that it's accurate is the huge value…that we're getting the right test result back to the right person. I suppose it’s brought some sort of consistency...maybe there's a bit of security, even, at knowing that it's online and it's going to be accurate, rather than a pen and paper… having something digital, I think is essential.”* – Site C.

Theme 6: internal policies and procedures:

- *“The issue is: How do we take the lessons that we've learned from this technical innovation and roll that into business as usual for future practice for the hospital? I'm sure there'll be a great deal more telehealth. I'm sure there'll be a big push towards getting a more formal customer relationship management application up - in fact, we just announced the team […] to create patient portals and to be able to get information back from patients in a more systematic way across a whole range of clinical domains. We're looking at how we can embed patient-generated health data into our EMR. So all of those things have been brought forward.”* – Site A.
- *“The nice thing about the REDCap program is it dumps it all into a database and you can export that in a variety of formats…*[such as] *for formal reporting. Also, when the patient's discharged, we have a summary of all of their data points throughout their journey and any sort of admissions to hospital, or complications.”* – Site C.
- *“There's no doubt, if we'd had a year to plan this, it would've been going through all sorts of IT and data security groups to think about these issues, and in fact, none of that happened.” –* Site C.

Theme 7: external policies and procedures:

- *“I think the COVID suite of solutions that we've employed are quite applicable and add immediate value to other hospitals that employ them. And that's proven via the handful of hospitals that have also deployed COVID-Care and COVID monitoring, COVID-Trace and the screening solutions. We've got a working group with a number of the other Victorian hospitals that are using the same solutions.”* – Site A.
- *“There's definitely space for using these tools to better coordinate our current management across our entire region; [..] deploying these tools to other sites and other healthcare services so that we then can basically allow our local public health unit to have a really detailed view over everybody in the area. Previously, one of the limiting factors is that [the state government] has not had great detail [about] the clinical progress of COVID patients and close contacts in the community. This is, I think, something that our tools do really well…pull that data in real time and have it visible to lots of different people if need be, and then be able to tailor it to the individual so that we are presenting the right data to the right people.”* – Site A*.*
- *“…We're thinking about some of this response to surges. Not just COVID, but other types of surges [e.g.] thunderstorm asthma, or if there was an explosion at a football stadium, you might get some serious injuries, but you might also get hundreds of relatively well people turning up. And could we use a similar system whereby we establish a clinic, direct all the relatively well people through it, [and] pull out the people who are sicker. We can give some information using QR codes. And so trying to set this up for the future, whereby you need to have it already kind of operational, but just sitting there (in hibernation)…You've got to pre-plan all of this. We think the government would be very excited by this, and we think other sites would probably be quite interested.”* – Site C.
